# Nutcracker syndrome; a rare cause of hematuria

**DOI:** 10.15171/jnp.2016.27

**Published:** 2016-07-29

**Authors:** Azar Nickavar

**Affiliations:** Department of Pediatric Nephrology, Iran University of Medical Sciences, Tehran, Iran

**Keywords:** Hematuria, Nutcracker syndrome, Children

**Implication for health policy/practice/research/medical education:**

Gross hematuria is an important concern in pediatric nephrology. Delayed diagnosis and treatment of the underlying pathology might predispose to irreversible consequences and chronic kidney disease. Recognition of all responsible etiologies are necessary to prevent unrelated examinations and delayed management.

Hematuria, especially in the gross from is a worrisome symptom for patients and their caregivers. Identification the etiology of hematuria may not be straightforward, and needs to multiple diagnostic approaches, especially in young children. Urinalysis, urine culture, measurement of urine protein and crystals are the primary simple and fast diagnostic tests for diagnosis of hematuria, followed by radiologic exams and renal biopsy in complicated patients. However, there are some rare cause of gross hematuria including nutcracker syndrome (NCS) with occasionally delayed and misdiagnosed, because of different manifestations and absence of consensus on diagnostic criteria (1,2).

The NCS or left venous hypertension is a rare clinical manifestation often caused by the compression of left renal vein (LRV) between abdominal aorta and superior mesenteric artery (SMA), an anatomical variation known as the nutcracker phenomenon (3).

Since its description in 1972, the aortomesenteric renal vein entrapment has been considered a rare cause of hematuria (4), with impaired blood outflow, renal venous hypertension and development of collateral circulation (5). Hematuria might occur secondary to the rupture of septum separating veins from the collecting system (4). LRV entrapment divides into anterior and posterior type, in which posterior and right-sided NCS are rare conditions (6).

NCS usually present in women and children (3), ranging from asymptomatic to variable symptoms including recurrent macroscopic hematuria with isomorphic urine red blood cells, left flank pain, pelvic congestion, orthostatic intolerance, and unilateral proteinuria without renal impairment (6-8), which may aggravate by exercise (4).

Early diagnosis is important to avoid unnecessary diagnostic procedures and complications such as LRV thrombosis and damage to the left kidney in untreated patients (9,10). Clinical manifestations are the main diagnostic options (6) and no validated diagnostic criteria exist (11).

Several imaging methods such as Doppler ultrasonography, computed tomography angiography, magnetic resonance angiography and retrograde venography (6) with measurement of pressure gradient between LRV and inferior vena cava (10) and hilar-aortomesenteric LRV diameter ratio >4 (12) are used to diagnose NCS. Observation of collateral veins in abdominal CT of asymptomatic patients may be optional for diagnosis of nutcracker phenomenon (13). Doppler examination is the diagnostic screening method for NCP (14).

Management of NCS depends on the clinical presentation, patient’s age and stage of the syndrome (6). There are several treatment options ranging from relatively long term conservative management to surgical procedures in patients with debilitating symptoms or refractory anemia (11,15).

Surgical procedures such as Gortex graft vein interposition, nephropexy, LRV endovascular stent placement, renal vein transposition and kidney autotransplantation are corrective surgical procedures with cessation of gross hematuria in almost all patients. However, complications such as retroperitoneal hematomas, stent migration, thrombosis and restenosis might occur after surgical management (3,10).

Associations such as SMA syndrome with gastrointestinal obstruction (16), and IgM nephropathy (17) have been reported in NCS.

In this paper a case of 12-year-old girl with rare association of nutcracker and Alport syndrome is presented who admitted for persistent severe microscopic hematuria, recurrent gross hematuria, occasional vomiting, loin pain and positive family history of microscopic hematuria associated with hearing deficit. Renal biopsy showed mild mesangial hyperplasia, negative immunofluorescence, splitting and irregular basement membrane thickness, in favor of Alport syndrome. However, abdominal CT scan showed classical right renal vein entrapment between abdominal aorta and SMA. Therefore, it is recommended to consider NCS in all children with recurrent macroscopic hematuria.

## Conflicts of interest

The author declared no competing interests.

## Author’s contribution

AN was the single author of the manuscript.

## Funding/Support

None
